# Enzymes from Fungal and Plant Origin Required for Chemical Diversification of Insecticidal Loline Alkaloids in Grass*-Epichloë* Symbiota

**DOI:** 10.1371/journal.pone.0115590

**Published:** 2014-12-22

**Authors:** Juan Pan, Minakshi Bhardwaj, Padmaja Nagabhyru, Robert B. Grossman, Christopher L. Schardl

**Affiliations:** 1 Department of Plant Pathology, University of Kentucky, Lexington, Kentucky, United States of America; 2 Department of Chemistry, University of Kentucky, Lexington, Kentucky, United States of America; University of Nebraska-Lincoln, United States of America

## Abstract

The lolines are a class of bioprotective alkaloids that are produced by *Epichloë* species, fungal endophytes of grasses. These alkaloids are saturated 1-aminopyrrolizidines with a C2 to C7 ether bridge, and are structurally differentiated by the various modifications of the 1-amino group: -NH_2_ (norloline), -NHCH_3_ (loline), -N(CH_3_)_2_ (*N*-methylloline), -N(CH_3_)Ac (*N*-acetylloline), -NHAc (*N*-acetylnorloline), and -N(CH_3_)CHO (*N*-formylloline). Other than the LolP cytochrome P450, which is required for conversion of *N*-methylloline to *N*-formylloline, the enzymatic steps for loline diversification have not yet been established. Through isotopic labeling, we determined that *N*-acetylnorloline is the first fully cyclized loline alkaloid, implying that deacetylation, methylation, and acetylation steps are all involved in loline alkaloid diversification. Two genes of the loline alkaloid biosynthesis (*LOL*) gene cluster, *lolN* and *lolM*, were predicted to encode an *N-*acetamidase (deacetylase) and a methyltransferase, respectively. A knockout strain lacking both *lolN* and *lolM* stopped the biosynthesis at *N*-acetylnorloline, and complementation with the two wild-type genes restored production of *N*-formylloline and *N*-acetylloline. These results indicated that *lolN* and *lolM* are required in the steps from *N*-acetylnorloline to other lolines. The function of LolM as an *N*-methyltransferase was confirmed by its heterologous expression in yeast resulting in conversion of norloline to loline, and of loline to *N*-methylloline. One of the more abundant lolines, *N*-acetylloline, was observed in some but not all plants with symbiotic *Epichloë siegelii*, and when provided with exogenous loline, asymbiotic meadow fescue (*Lolium pratense*) plants produced *N*-acetylloline, suggesting that a plant acetyltransferase catalyzes *N*-acetylloline formation. We conclude that although most loline alkaloid biosynthesis reactions are catalyzed by fungal enzymes, both fungal and plant enzymes are responsible for the chemical diversification steps *in symbio*.

## Introduction

**Figure 1:**
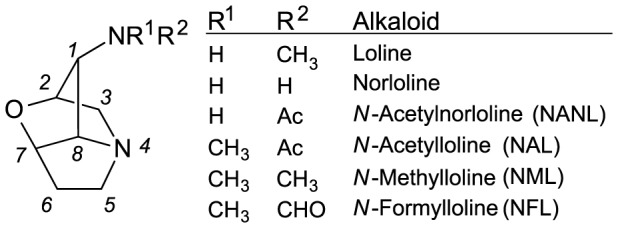
Structures of common loline alkaloids. Substitutions on the nitrogen at C1 differentiate the lolines.

Many cool-season grasses (Poaceae, subfamily Poöideae) are infected by endophytic fungi of genus *Epichloë*. The endophyte systemically colonizes aboveground tissues of the grass and grows along the longitudinal axis between plant cells. Many *Epichloë* species rely completely on the host for dissemination through the seeds, whereas some are additionally or solely transmitted horizontally to new plants via sexual spores. Although the horizontally transmitted species are somewhat pathogenic — causing choke disease in forming their sexual reproduction structures (stromata) — all *Epichloë* species spend most or all of their life cycle in plant intercellular spaces and cause no obvious harm to their hosts. This close interaction of the two organisms tends to foster mutualistic interaction in which the endophyte helps to defend its host grass against invertebrate or vertebrate herbivory through production of various kinds of alkaloids. Four classes of alkaloids are produced by these endophytic fungi — ergot alkaloids, lolitrems, peramine, and loline alkaloids — all of which confer herbivory resistance [1]. The loline alkaloids specifically deter invertebrate herbivores [1], [2]. Lolines found in grass-*Epichloë* symbiota (Fig. 1) differ in modifications of the 1-amino group: -NH_2_ (norloline), -NHCH_3_ (loline), -N(CH_3_)_2_ (*N*-methylloline  =  NML), -N(CH_3_)Ac (*N*-acetylloline  =  NAL), -NHAc (*N*-acetylnorloline  =  NANL), and -N(CH_3_)CHO (*N*-formylloline  =  NFL).

The asexual seed-transmitted *Epichloë* species are often found to be inter- or intra-species hybrids [3]–[5], which (among other benefits) can pyramid multiple alkaloid biosynthesis gene clusters and promote diversification of alkaloid profiles [6], [7]. A likely benefit of diversifying defensive alkaloids is to reduce the potential for herbivores to develop resistance, provided that different alkaloids have different modes of specificity [8]. Another potential advantage is that different alkaloids may affect different herbivores and hence broaden the spectrum of host protection. The ability of grass-*Epichloë* symbiota to produce a variety of protective alkaloids greatly enhances the plant-defensive arsenal [9]. Even among loline alkaloids, differently modified forms have been reported to exert different effects on insects [10]. For example, at high concentrations (800 and 1600 µg/g), NFL reduces the growth and development of Argentine stem weevil (*Listronotus bonariensis*) larvae, whereas NANL causes high mortality of Argentine stem weevil larvae but with little effect on the growth or development of the larvae [11]. These interesting observations raise the question of how the diverse loline alkaloids are synthesized and how profiles of different loline alkaloids have evolved.

Several genomes of loline alkaloid-producing *Epichloë* species have been sequenced, and a *LOL* cluster that encodes enzymes involved in loline alkaloid biosynthesis has been found in all of them. The endophytes that produce NFL together with the other loline alkaloids have a total of 11 *LOL* genes positioned in the cluster in the order *lolF*, *lolC*, *lolD*, *lolO*, *lolA*, *lolU*, *lolP*, *lolT*, *lolE*, *lolN* and *lolM* [6]. Others that produce NANL or the bicyclic *exo*-1-acetamidopyrrolizidine (AcAP) as the end product have subsets of the 11 aforementioned genes, either lacking genes or having inactivating mutations in late pathway genes [6]. Functions of enzymes encoded by *LOL* genes have been suggested based on bioinformatic analysis [12]. LolC has been proposed to catalyze an unusual γ-substitution reaction that condenses proline and homoserine, and it has been established to be involved in the pathway by an RNA-interference (RNAi) experiment [13]. A predicted cytochrome P450 enzyme, LolP, is required for oxygenation of NML to produce NFL and is not involved in earlier biosynthetic steps [14]. Recently, it has been established that ether-bridge formation requires the 2-oxoglutarate-dependent non-heme iron oxygenase, LolO [15]. The other loline alkaloid diversification steps have not yet been identified. Here we establish the basis for the diversity of loline alkaloids, demonstrating that LolN and LolM are required for biosynthetic steps from NANL to NML, and also showing that the host plant converts loline to NAL.

## Materials and Methods

### Biological materials and general experimental procedures

Fungal strains (Table 1) were isolated and cultured on potato dextrose agar (PDA) at 22°C as previously described [16]. Infection of the grass with or without the endophyte was checked by tissue-print immunoblot [17], then grow-out of the fungus from surface-sterilized grass tissue, and confirmation of the endophyte strain using polymerase chain reaction (PCR) with specific primers. General experimental procedures and reagents were as previously reported [15]. Primers used in this study are listed in S1 Table.

**Table 1:** Fungal isolates in this study.

Fungus	Isolate	Host	Origin
*Epichloë festucae*	E2368	*Lolium s*pp.	Lexington, Kentucky, USA
*Epichloë uncinata*	e167	*Lolium pratense*	Nyon, Switzerland
*Epichloë coenophiala*	e19	*Lolium arundinaceum*	Lexington, Kentucky, USA
*Epichloë amarillans*	E57	*Agrostis hiemalis*	Brazoria Co., Texas, USA
*Epichloë glyceriae*	E2772	*Glyceria striata*	Canastota, New York, USA
*Epichloë canadensis*	e4815	*Elymus canadensis*	Throckmorton County, Texas
*Epichloë canadensis*	e4814	*Elymus canadensis*	Nuevo León State, Mexico
*Epichloë brachyelytri*	E4804	*Brachyelytrum erectum*	Edmonson Co., Kentucky, USA
*Epichloë amarillans*	E722	*Sphenopholis obtusata*	Georgia, USA
*Atkinsonella hypoxylon*	B4728	*Danthonia spicata*	Lexington, North Carolina, USA
*Epichloë siegelii*	ATCC 74483	*Lolium pratense*	PI 237707, Western Regional Plant Introduction Station, Pullman Washington, USAUSA
*Epichloë coenophiala*	e4309	*Lolium arundinaceum*	PI 598903, Western Regional Plant Introduction Station
*Epichloë coenophiala*	e4163	*Lolium arundinaceum*	PI 422777, Western Regional PlantIntroduction Station Station
*Epichloë sp.*	E4686	*Poa autumnalis*	Texas, USA
*Epichloë* sp.	e4779	*Festuca versuta*	Taber, Austin, Texas, USA

Loline alkaloids were extracted with chloroform, using quinoline as internal standard, and analyzed by GC-MS as previously reported [18]. Extraction from plant and minimal-medium cultures followed previously reported methods [15].

### Preparation of tetradeuterated AcAP

This procedure was conducted as previously reported for preparation of AcAP [15], except that we used (±)-3,3-dideutero-1-oximinopyrrolizidine [18] as the starting material to label the pyrrolizidine ring on C3, and we used [^2^H_6_]-acetic anhydride to add a trideuteroacetyl group to the *exo*-1-aminopyrrolizidine. The resulting products were 13.5% *d*_3_-, 85% *d*_4_-, and only trace amounts of *d*_5_-*exo*-1-acetamidopyrrolizidine. Substitution of deuterium with protium from H_2_O must have occurred during the Raney Ni reduction, rendering *exo*-1-trideuteroacetamido-3-deuteropyrrolizidine ([^2^H_4_]AcAP) as the major product. (In fact, washing the Raney Ni with D_2_O before adding the oxime yielded products with up to seven D atoms incorporated.) Fortunately, this less highly deuterated material remained suitable for the experiments for which it was intended.

### Application of [^2^H_4_]AcAP to *Epichloë uncinata* e167 culture

*Epichloë uncinata* e167 was grown in minimal medium to induce loline-alkaloid production as described previously [19]. The labeled compound was applied at the onset of loline alkaloid production (9^th^ day post inoculation) at a final concentration of 3.75 mM. L-[U-^2^H_7_]Pro (4 mM) or Pro (4 mM) was applied following the same procedure as control. To each of the treatments DMSO (1.5%) was also added to increase permeability of the cells. The cultures were shaken at 55 rpm, 22°C, for another 35 days and then checked for loline alkaloids.

### Construction of gene-replacement and complementation plasmids

In *Epichloë festucae* E2368, *lolN* and *lolM* are adjacent and divergently transcribed. A plasmid was constructed to replace *lolM* and most of *lolN* in *E. festucae* E2368 with a modified *hph* marker gene [20] via homologous recombination. Using E2368 genomic DNA as the template, a 2.5-kb fragment upstream of the target *lolN*-*lolM* segment was amplified by PCR with primers upFLKf (*Xba*I) and upFLKr (*Xba*I), digested with *Xba*I, and ligated with pKAES173 [14] digested with *Xba*I. The ligated product was introduced by transformation into *Escherichia coli* XL1-blue competent cells, and transformants were screened for *hph* by colony-PCR with primers hphf and hphr. Positive colonies were extracted for plasmid DNA, which was then tested by restriction-endonuclease digestions and Sanger sequencing to identify the construct that had the correct orientation. The proper construct was then digested with *Xma*I and *Xho*I. A 1.9-kb fragment downstream of the *lolN*-*lolM* segment was amplified by PCR with primers lolAANMTdf (*Xma*I) and lolAANMTdr (*Xho*I), and then digested with *Xma*I and *Xho*I. The digested plasmid and PCR product were gel purified and ligated to generate plasmid pKAES323, which has flanking sequences of the target *lolN*-*lolM* region on either side of the *hph* expression construct.

To complement the *lolN* and *lolM* double-knockout strain, *lolN* and *lolM* of E2368, together with their common, divergent promoter, were amplified by PCR with primers upFLKf and lolNMkops using Phusion Hot Start High-Fidelity DNA Polymerase (Thermo Scientific, Ratastie, Vantaa, Finland) with HF buffer (with 1.5 mM MgCl_2_) provided from the manufacturer. The temperature conditions were 98°C for 3 min, 35 cycles of 98°C for 10 s, 62°C for 10 s, and 72 for °C 3 min, then a final 5 min incubation at 72°C. The PCR product was purified and digested with *Xba*I. Plasmid pKAES215 (S1 Protocol) was digested with *Xba*I and *Eco*RV. The two fragments were gel purified and ligated to generate pKAES341, which confers hygromycin resistance and expresses *lolN* and *lolM* driven by their native, divergent promoter. All plasmid constructs were confirmed by Sanger sequencing.

### Fungal transformation and screening for knockouts

Protoplast preparation and transformation procedures were performed as previously described [21] with minor changes [15]. After electroporation, *lolN* and *lolM* knockout transformants of E2368 were selected on regeneration medium with 450 µg/ml hygromycin B. The same selection was also used in single spore isolation and fungal isolate maintenance on PDA. After two single-spore isolations, the transformants were extracted for DNA with DNeasy 96 Plant Kit (Qiagen, Valencia, CA, USA) and screened for the *lolN* gene by PCR with primers aamAup1 and aamAd1. The transformants that were negative in the *lolN* screening were then positively screened with primers upFLKf and lolNMkops, which produced a 4.0 kb product from the knockout transformant and a 5.7 kb product from the wild-type strain. As controls, an ectopic transformant with pKAES323 and an empty-vector transformant with plasmid pKAES173 were also generated and confirmed by PCR.

Transformation of the knockout strain with pKAES341 for complementation of *lolN* and *lolM* was performed by co-transforming linearized pKAES341 and pII99, which confers geneticin resistance [22], at a molar ratio of 5∶1. Selection of transformants and single-spore isolations were carried out on PDA plates with 800 µg/ml G418. The transformants were single-spore isolated twice and extracted for DNA to screen for *lolN* by PCR with primers aamAup1 and aamAd1, and for *lolM* with primers aamAup1 and lolNMkops.

Inoculation of the strains to endophyte-free meadow fescue was carried out as described by Latch and Christensen [23]. Loline-alkaloid profiles of the plants were checked approximately 6 months after inoculation.

### Southern-blot analysis

Genomic DNA was digested with *Hin*dIII and 1 µg of each DNA was electrophoresed in 0.7% agarose gel (Phenix Research, Candler, NC, USA) in 0.5 x TBE buffer [44.5 mM tris(hydroxymethyl)aminomethane (Tris) base, 44.5 mM boric acid, 1 mM ethylenediaminetetraacetic acid (EDTA), pH 8.2] for 20 hr in a 4°C cold room. The DNA was then transferred to Hybond H^+^-nylon membrane (GE Healthcare, Piscataway, NJ, USA) with a GENIE electroblotter (Idea Scientific, Minneapolis, MN, USA). Immobilized DNA on the membrane was denatured in 0.4 M NaOH for 10 min and neutralized in 2x SSC for 10 min. The DNA was cross-linked to the membrane with UV light using a Spectrolinker (Spectronics, Westbury, NY, USA). Probes for *lolN* and *lolM* were prepared by PCR with primers Nexpf and Nexpr, and Mexpf and Mexpr, respectively, using E2368 DNA as template. A 974-bp PCR product for *hph* probe was prepared by PCR with primers hphf and hphr using pKAES173 as template. The PCR products were purified and labeled with α-[^32^P]dCTP (GE Healthcare) using the Prime-a-Gene Labeling System (Promega, Madison, WI, USA). Hybridization was performed as in Starnes et al. [24]. Hybridizations of probes for *lolN* and *lolM* were performed sequentially on the same membrane, whereby the probe from each previous hybridization was removed by incubating the membrane twice with 0.4 M NaOH for 30 min at 45°C, followed by washing with 0.1x SSC 0.1% SDS for 15 min at 45°C. Subsequent hybridization to an *hph* probe was used to verify the quality and approximately equal quantities of the DNA in each lane.

### Yeast expression of *lolM* and western blot analysis

The full-length *lolM* was amplified from cDNA prepared from mRNA of *E. uncinata* e167 that was induced to produce lolines by ca. 20 day culture in minimal medium [19]. Primer pair MexpYNfBglII and MexpYNrBglII was used to amplify *lolM* with *Bgl*II incorporated in the 5′ ends so that *lolM* would be expressed with an N-terminal FLAG tag [25]. The PCR products were purified and digested with *Bgl*II, and ligated with *Bgl*II-digested pESC-LEU (Agilent Technologies, Santa Clara, CA, USA). Resulting plasmids with the correct orientation were chosen through endonuclease digestion and further confirmed by Sanger sequencing. The resulting plasmid pKAES350 has *lolM* under control of the promoter *GAL10*, with an N-terminal FLAG tag. Yeast (*Saccharomyces cerevisiae*) strain YPH499 (MATa *ura3*-52 *lys2*-801^amber^ *ade*2-101^ochre^ *trp*1-Δ63 *his*3-Δ200 *leu*2-Δ1) [26] (Agilent Technologies) was used to express *lolM*. Yeast competent cell preparation and transformation were performed according to the manufacture's protocol (Agilent Technologies), and transformants were selected on synthetic dextrose minimal medium without leucine (SD/-Leu) agar plate. The growing colonies were then streaked twice on the same medium before feeding experiments.

Expression of the LolM protein was confirmed by western blot analysis as follows. Yeast crude protein was extracted following the methods described in Rajendran et al. [27]; then the proteins were fractioned by electrophoresis in a SDS-PAGE gel (10%). The primary antibody was α-FLAG (Sigma-Aldrich, St. Louis, MO), and immunoblots were developed using an ECL detection kit (Roche Applied Science, Basel, Switzerland).

### Application of norloline or loline to yeast crude protein extract

Yeast transformed with pKAES350 or empty vector pESC-LEU were grown in SD/-Leu broth at 30°C overnight, washed twice with synthetic galactose minimal medium without leucine (SG/-Leu), and resuspended in SG/-Leu medium to OD_600_ of 0.5. The cultures were grown at 30°C, 250 rpm for 2 days before sampling. The yeast cells were centrifuged at 1000×*g* for 5 min, and the cell pellet was weighed. Approximately 300 mg of yeast pellet was used for each crude protein extraction following the procedure described in [28] with modifications. A Fastprep machine (20 sec at 4 m/s, repeated four times) was used to break the cells in a Tris-HCl buffer (50 mM Tris-HCl, pH 7.5, 10% glycerol, 10 mM dithiothreitol), with proteinase (cOmplete ULTRA Tablets, Mini, EDTA-free, Roche) added right before extraction. Either loline or norloline (4 mM), and *S*-adenosyl methionine (AdoMet) (1 mM), were added to 90 µl of the crude extract for the enzyme assay. Crude protein extract with loline or norloline but without adding AdoMet was also assayed as a check that the reaction requires AdoMet. The assay mixture was incubated at 30°C overnight, and the reaction was stopped by adding 100 µl of 1M NaOH. Then the loline alkaloids were extracted with chloroform and analyzed by GC-MS.

Loline used in this study was prepared from endophyte infected tall fescue seeds according to the previously reported procedure (Petroski et al., 1989). Norloline was prepared from *Adenocarpus decorticans* seeds (100 g). The ground seeds were basified by adding 1 M NaOH to pH 11, then extracted with chloroform (5 x, 10 ml/g seed). Then the alkaloid mixture was concentrated by rotary evaporation, during which the loline alkaloids were hydrolyzed to norloline due to concentration of the base. Then the mixture was applied on silica gel column (chloroform: methanol: NH_4_OH  = 49.5: 49.5: 1 as the running solvent) to purify approximately 10 mg of norloline.

### Genome sequencing and phylogenetic analysis

Fungal DNA was isolated and sequenced by pyrosequencing on a Roche/454 GS FLX+ platform, and assembled as previously described [6], [7]. Genes were annotated as previously described [6] from sequenced genomes of *Epichloë* species and *Atkinsonella hypoxylon*, available at www.endophyte.uky.edu and the National Center for Biotechnology Information. The *lolN* sequences of *Epichloë* sp. e4686 and e4779 were obtained by PCR with primers Nexpf and lolNampr and sequencing of the PCR products. The sequences were deposited in GenBank with accession numbers KJ653809 for e4686 *lolN* and KJ653810 for e4779 *lolN*. Alignment of DNA sequences and amino acid sequences was performed on www.phylogeny.fr [29], and edited with MacVector v. 12.7.5.

## Results and Discussion

### Retention of the deuterium atoms from [^2^H_4_]AcAP in loline alkaloid product

**Figure 2:**
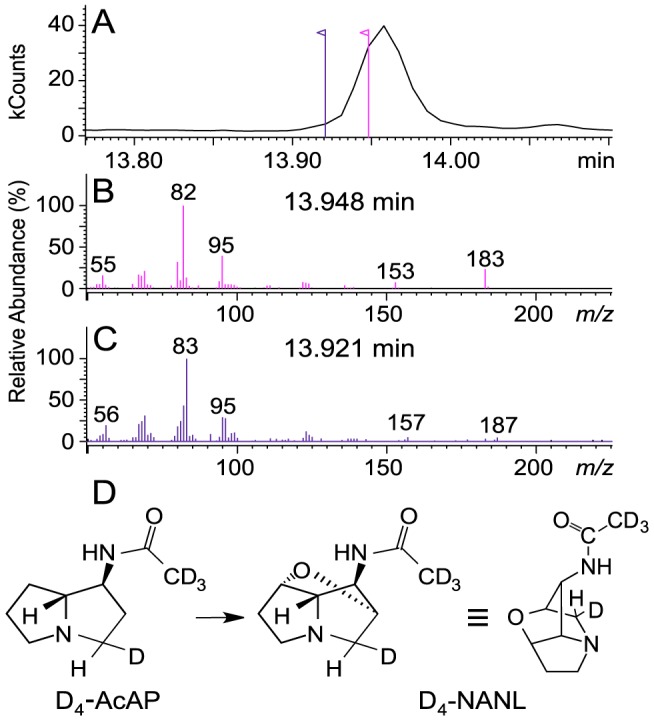
Enrichment of deuterated *N*-acetylnorloline (NANL) from application of tetradeuterated *exo*-1-acetemidopyrrolizidine ([^2^H_4_]AcAP) to loline alkaloid producing culture. Shown are (A) GC-MS total ion chromatogram, (B) mass spectrum at retention time 13.948 and (C) 13.921 min, and (D) proposed scheme of [^2^H_4_]NANL formation from [^2^H_4_]AcAP.

A previous study showed that LolO is required in formation of the ether bridge of loline alkaloids [15], but it did not determine whether AcAP is the direct precursor of NANL. An alternative hypothesis is that *exo*-1-aminopyrrolizidine (1-AP) is the real precursor to be oxidized to form norloline, whereas AcAP and NANL are both shunt products generated by acetylation of 1-AP and norloline, respectively. To test these alternative possibilities, we prepared [^2^H_4_]AcAP with one deuterium atom at C3 of the pyrrolizidine ring system and three deuterium atoms on the acetyl group, and we applied it to a culture of *E. uncinata* e167 under loline alkaloid-production conditions [19]. If the acetylation of 1-AP occurred before ether-bridge formation, we expected NANL to retain four deuterium atoms, whereas if AcAP were first deacetylated to 1-AP before ether-bridge formation, we expected it to retain only one deuterium. In the event, we observed a compound that was determined by MS to be tetradeuterated NANL at the front edge of the NANL peak, with parent ion *m/z*  = 187 (*m/z*  = 183+4), and major fragment ions with *m/z*  = 83 (*m/z*  = 82+1) and *m/z*  = 157 (*m/z*  = 153+4) (Fig. 2). This result clearly demonstrated the linear nature of the biosynthetic pathway and established AcAP and NANL as true intermediates. Hence, the likely pathway for the diversification of loline alkaloids from NANL would involve deacetylation, methylation, and acetylation.

### Deletion of the *lolN* and *lolM* genes resulted in accumulation of NANL and eliminated NFL production

**Figure 3:**
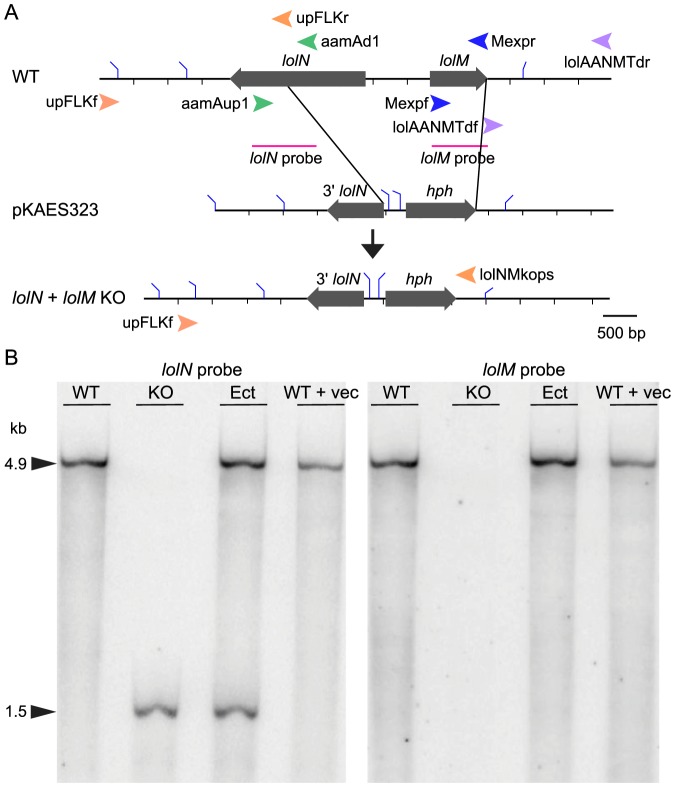
Replacement of *lolN* and *lolM* with *hph* maker gene. (A) Schematic representation of *lolN*-*lolM* replacement by the *hph* marker gene via homologous recombination. Shown are maps of the wild-type *lolN* and *lolM* in *Epichloë festucae* E2368 (WT), targeting vector (pKAES323), and the locus after homologous recombination (KO). Black bars represent DNA sequence, and filled arrows represent genes. Bent blue lines on the bars represent *Hin*dIII digestion sites. Colored arrowheads represent primers used to generate pKAES323 and to screen the transformants. (B) Southern-blot analysis of *E. festucae* strains. Wild-type E2368 and transformants were probed with a *lolN* fragment or *lolM* gene amplified from E2368 (old probe was stripped off the membrane before new hybridization). Lanes contained *Hin*dIII-digested genomic DNA from E2368 (WT), *lolN*-*lolM* knockout transformant (KO), ectopic transformant of E2368 with pKAES323 (Ect), and E2368 transformed with the empty vector pKAES173 (WT+vec).

The *lolN* and *lolM* genes are found in all NFL-producing *Epichloë* species, and in the genome assembly of *Epichloë festucae* E2368 they are linked with the known *LOL* cluster [6]. Bioinformatic analyses of these genes suggested that LolN is an acetamidase and LolM is a methyltransferase. The functions fit well into our hypothesis that NANL must be deacetylated to produce norloline, which is then methylated to form loline and NML, the precursor of NFL [14]. Hence, it appeared likely that LolN and LolM are involved in NFL biosynthesis from NANL. In *E. festucae* E2368, *lolN* and *lolM* share a common, divergent promoter, so in order to investigate functions of *lolN* and *lolM*, we constructed a plasmid to replace both genes with marker gene *hph* through homologous recombination (Fig. 3A).

**Figure 4:**
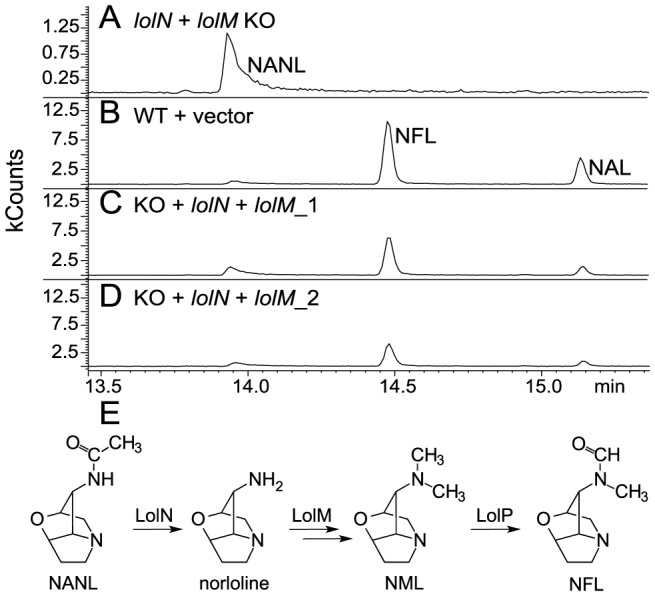
GC-MS traces showing loline-alkaloid profiles of meadow fescue symbiotic with different *E. festucae* strains. (A) The *lolN*-*lolM* knockout (KO), (B) an empty-vector control transformant (WT+vec), and (C and D) complementation strains (KO+*lolN*+*lolM*). The numbers after complementation strains represent different meadow fescue plants inoculated with independent transformants. (E) Proposed roles of LolN and LolM (this work), and reported role of LolP [14], in the biosynthetic pathway from *N*-acetylnorloline (NANL) to the final product, *N*-formylloline (NFL).

One *lolN* and *lolM* double-knockout transformant was obtained from 150 transformants. This knockout strain was confirmed by Southern-blot analysis to lack full-length *lolN* and *lolM* (Fig. 3B). The knockout strain, an ectopic transformant, and an empty-vector transformant were inoculated back into endophyte-free meadow fescue seedlings to check their loline-alkaloid profiles (*E. festucae* produces lolines only *in symbio*). Plants with the knockout mutant contained NANL as the only loline alkaloid detected, whereas plants with the control and wild-type strains contained NFL, NAL, and NANL (Fig. 4). To confirm that the change of loline alkaloid profile was due to loss of a functional copy of *lolN* and *lolM*, a complementation plasmid that had *lolN* and *lolM* under their native promoter was introduced into the knockout mutant. Three independent complementation strains were obtained and inoculated into meadow fescue to check the loline alkaloid profile. All three transformants restored production of NFL and NAL *in planta* (Fig. 4). These results indicate that *lolN* and *lolM* are required in the pathway from NANL to the biosynthetic end product, NFL.

### Natural NANL accumulators have mutations or deletions of *lolN*

If *lolN* and *lolM* are indeed involved in the production of NFL from NANL as we hypothesized, it is conceivable that endophytes that produce NANL as the end product may have mutations in or deletions of *lolN*. In order to test this hypothesis, *LOL* clusters of several loline-alkaloid producers were compared. Endophytes that showed one of the three chemotypes, NANL, AcAP, or NFL as the end product shared most of the *LOL* genes except for *lolO*, *lolP, lolN* or *lolM* (Table 2). Strains with mutations in, or complete lack of, *lolO* accumulated AcAP, consistent with the previously reported function of *lolO* [15]. Interestingly, these strains also lacked functional copies of *lolN*, *lolM*, and *lolP*. Strains that had a functional *lolO*, but lacked or had a mutated *lolN*, all accumulated NANL. Conversely, those strains from plants that accumulated NANL but not NFL, NML, or NAL had lost or obviously defective *lolN*, *lolM* and *lolP* genes, with the sole exception of *Epichloë coenophiala* strain e4309 (discussed next).

**Table 2:** Loline alkaloid profiles and *LOL-*gene screening results for endophyte isolates. a

	Alkaloids						*LOL* genes			
Endophyte isolates	AcAP	Loline	NANL	NAL	NML	NFL	*lolO*	*lolP*	*lolN*	*lolM*
E2368	tr	+	+	+	+	+	+	+	+	+
e167	tr	+	+	+	+	+	+	+	+	+
e19	tr	+	+	+	+	+	+	+	+	+
e4163	tr	+	+	+	+	+	+	+	+	+
E57	−	−	+	−	−	−	+	Ψ	−	−
E2772	−	−	+	−	−	−	+	−	Ψ	−
E4815	−	−	+	−	−	−	+	Ψ	−	−
e4309	−	−	+	−	−	−	+	+	+^b^	+
e4814	+	−	−	−	−	−	Ψ	Ψ	nd	nd
E4804	+	−	−	−	−	−	Ψ	−	Ψ	Ψ
E722	+	−	−	−	−	−	Ψ	−	Ψ	Ψ
B4728	+	−	−	−	−	−	Ψ	Ψ	−	−

a Abbreviations are: tr  =  trace amount; +  =  alkaloid detected or full gene present; −  =  alkaloid not detected or gene not present; Ψ  =  pseudogene; nd  =  gene not detected in PCR screen.

b Two nonsynonymous mutations found in otherwise conserved sites G495D and E551K.

**Figure 5:**
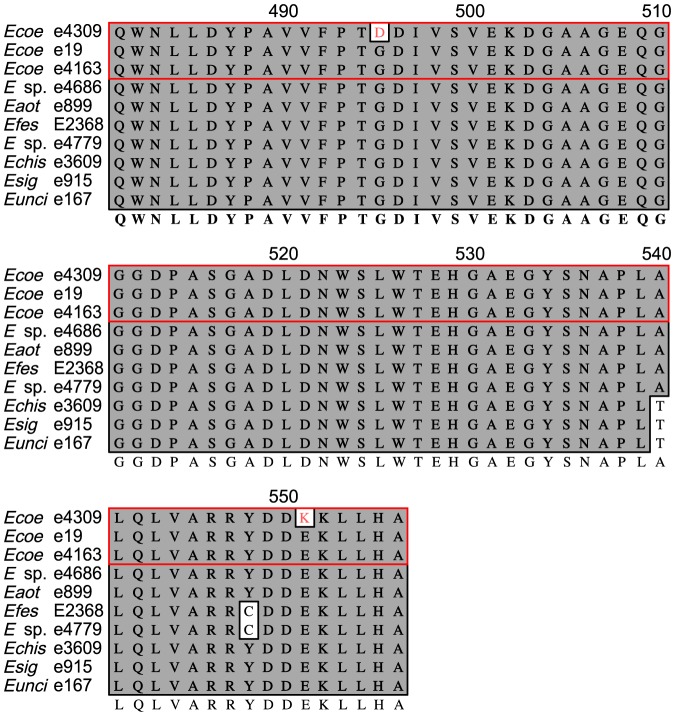
Partial LolN amino-acid sequence alignment of *Epichloë coenophiala* e4309 and *N*-formylloline (NFL) producers. Red-framed sequences are three different *E. coenophiala* isolates. *Ecoe*  =  *Epichloë coenophiala*, *Eaot*  =  *Epichloë aotearoae*, *Efes*  =  *Epichloë festucae*, *Echis*  =  *Epichloë chisosa*, *Esig*  =  *Epichloë siegelii*, *Eunci*  =  *Epichloë uncinata*.

In contrast to other NANL accumulators, strain e4309 had a *LOL* cluster with the complete set of 11 genes and no obvious inactivating mutation in any of them. Therefore, we aligned and compared *lolN* gene sequences and predicted amino acid sequences of e4309 to those of known NFL producers. LolN of e4309 had two nonsynonymous changes, G495D and E551K, compared to LolN of strains that produced NFL (Fig. 5). Moreover, when strain e4309 was compared to two other *E. coenophiala* strains that produce NFL (e19 and e4163), amino acid sequences of all *LOL* genes were identical except for the aforementioned G495D and E551K changes in *lolN* of e4309. Reverse transcription-PCR (RT-PCR) indicated that *lolN* and *lolM* were expressed by e4309 *in symbio*. Thus, one or both of the two alterations in LolN observed in e4309 might have rendered the protein dysfunctional for deacetylation of NANL.

Results from experiments with the *lolN* and *lolM* double knockout, and comparison of *LOL* clusters of various species and strains, consistently showed that NANL accumulation was associated with *lolN* dysfunction, supporting its predicted function as an acetamidase that would deacetylate NANL to form norloline in the biosynthetic pathway.

### LolM expressed in yeast catalyzed methylation of norloline and loline

**Figure 6:**
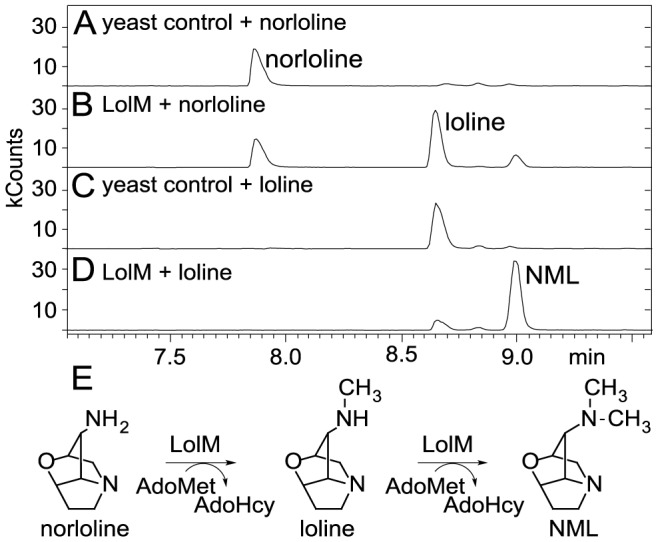
Assay of LolM methyltransferase activity. (A) Chromatogram of loline alkaloids from incubation of norloline and AdoMet with protein extract of yeast transformed with empty vector. (B) Chromatogram of loline alkaloids from incubation of norloline and AdoMet with crude protein extract from yeast that expresses LolM. (C) Chromatogram of loline alkaloids from incubation of loline and AdoMet with protein extract of yeast transformed with empty vector. (D) Chromatogram of loline alkaloids from incubation of loline and AdoMet with crude protein extract from yeast that expresses LolM. (E) Proposed scheme of loline and *N*-methylloline (NML) formation from norloline. AdoHcy  =  *S*-adenosyl homocysteine.

To test if the *lolM* gene product functions as a methyltransferase to methylate norloline in consecutive steps to form loline and NML, we expressed *lolM* in yeast. Crude protein extract from LolM-expressing yeast was prepared and incubated with AdoMet plus either loline or norloline. Loline and a smaller amount of NML were observed from application of norloline to the crude protein extract when incubated with AdoMet (Fig. 6). Extract of yeast transformed with the empty vector failed to catalyze methylation of norloline. Similarly, the protein extract from LolM-expressing yeast catalyzed the conversion of almost half of the added loline to NML, whereas no increase in NML was observed after incubating loline and AdoMet with protein extract from yeast transformed with the empty vector (Fig. 6). These results demonstrated that LolM was responsible for methylation of norloline to loline, and loline to NML.

### Plant acetyltransferase responsible for NAL production

The acetylated form of loline, *N*-acetylloline (NAL), is also a common loline alkaloid observed in many infected grasses together with NANL and NFL. It has been reported that symbiota comprising the endophyte species FaTG-3 and its natural host, tall fescue (*Lolium arundinaceum*), contain NFL and NAL, but when FaTG-3 is inoculated to perennial ryegrass (*Lolium perenne*), the resulting symbiota contain NFL, but lack NAL [30]. Furthermore, we observed that tall fescue and meadow fescue symbiotic with *Epichloë siegelii* accumulated NFL, NANL, and NAL, whereas some perennial ryegrass plants symbiotic with the same fungus contained NFL and NANL, but lacked NAL, indicating that plant genotype affects production of NAL. In addition, no NAL was detected in loline-alkaloid producing cultures of *E. uncinata* e167, consistent with a previously reported observation [19], whereas meadow fescue symbiotic with e167 possessed NAL and other loline alkaloids. A culture of e167 sometimes gave a small peak of similar *m/z* as NAL, which migrated in GC near, but not coincident with, NAL, when compared to extracts from meadow fescue-*E. uncinata* e167 symbiota (S1 Fig.). We judged this peak as probably representing *N-*propionylnorloline (also known as decorticasine) based on comparison with extracts from *Adenocarpus decorticans*, in which decorticasine is the major pyrrolizidine alkaloid [31], [32].

**Figure 7:**
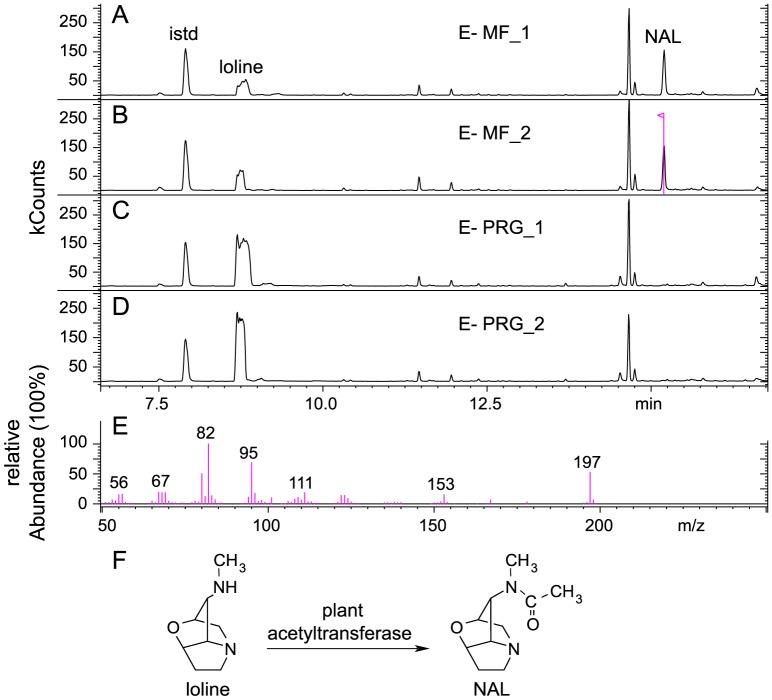
GC-MS chromatogram of loline alkaloids after application of loline to asymbiotic plants. Shown are loline alkaloids extracted from loline applications to (A and B) endophyte-free (E-) meadow fescue (MF), and (C and D) E- perennial ryegrass (PRG), (E) mass spectrum of *N*-acetylloline (NAL) from application of loline to E- meadow fescue, and (F) proposed scheme of NAL formation from loline. Quinoline was added as internal standard (istd). Unlabeled peaks are non-loline alkaloid compounds. Numbers after MF or PRG indicate independent trials.

We hypothesized that a plant enzyme is required to acetylate loline to form NAL. To test this hypothesis, loline was applied to asymbiotic meadow fescue and perennial ryegrass, and the alkaloid profile of the symbiota was analyzed. Compound NAL was observed in extracts from loline-fed meadow fescue, but not from loline-fed perennial ryegrass (Fig. 7), suggesting that a grass acetyltransferase enzyme catalyzes the conversion of loline to NAL. This result was surprising, considering that most of the known loline biosynthetic pathway is catalyzed by enzymes encoded in the fungal *LOL* cluster [14], [15], [33]. This interesting finding raises questions for future investigations, such as the following: Does the conversion of loline to NAL occur within or outside of the plant cells? What is the evolutionary origin of this plant acetyltransferase? Is it specific for NAL production, or does it catalyze other acetylation reactions as well? Is there genotypic variation for NAL production within grass species? Are there other metabolic pathways with both fungal and plant components?

An acetyltransferase is also needed to produce AcAP from 1-AP, but this enzyme does not seem to be of plant origin, because AcAP is produced in *E. uncinata* cultures outside of the plant system. It will be intriguing to learn how the two partners of the symbiosis communicate with each other and regulate the possible trafficking of loline alkaloids between them.

### Evolution and implications of loline alkaloid diversity

**Figure 8:**
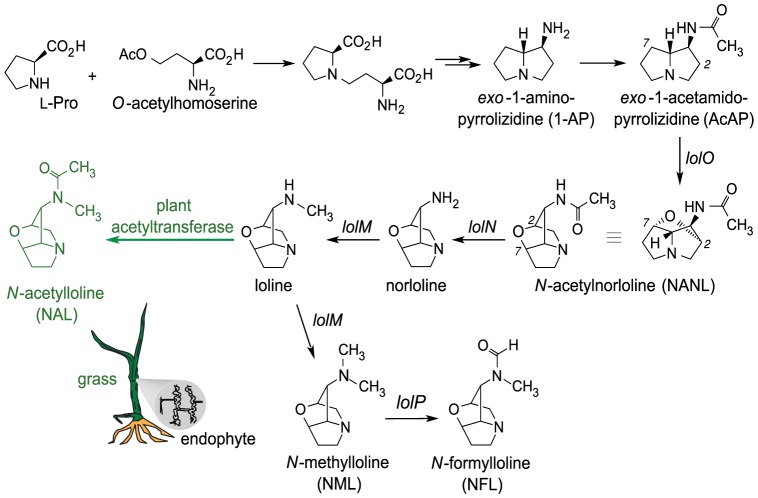
Summarized loline-alkaloid biosynthetic pathway. Labeled arrows are for steps that contribute to diversity of the lolines. Presence or absence of functional copies of *lolO, lolN, lolM*, or *lolP*, or a plant acetyltransferase activity, determine which alkaloids accumulate in the symbiotic plant as the pathway end-products.

Alkaloid profiling showed that *Epichloë* species produced different loline alkaloids depending on the gene content of their *LOL* clusters. Fig. 8 summarizes current knowledge of the biosynthetic pathway, and indicates the genes that, by their presence or absence, determine loline alkaloid diversity. When there was a full complement of the 11 *LOL* genes, NFL, NAL, and NANL were produced *in symbio*, but when there was mutation in or deletion of *lolO* or *lolN*, the accumulated end-product was AcAP or NANL, respectively (Table 1). Remnants of *lolN*, *lolO*, *lolM*, or *lolP* were often found in strains with NANL or AcAP as end-products, indicating that evolution of these strains involved loss of genes from the ancestral 11-gene *LOL* cluster. In strains for which NANL was the end product, *lolN* genes showed nonsynonymous point mutations (e4309), a partial deletion (E2772), or complete absence (E57 and e4815). Among strains with AcAP end-product, *lolO* had been inactivated by apparently independent mutations [15]. The multiple inactivations and losses of late pathway genes that alter loline alkaloid profiles suggest that loline alkaloid production may be under diversifying selection.

We also found that the plant can directly contribute to diversity of these fungal metabolites. An acetyltransferase activity associated with the host plant is responsible for conversion of loline to NAL, adding a layer of chemotypic complexity to this close fungus-plant symbiotic interaction. Although it is well established that symbiotic organisms may be interdependent in production of indispensible metabolites [34], we are unaware of any other report of one partner modifying specialized (secondary) metabolites produced by the other in a mutualistic symbiosis.

Loline alkaloids show broad-spectrum anti-insect activity, and different lolines may have different biological activities. *In vitro* tests of NFL, NAL, NML, and semisynthetic loline derivatives with long carbon-chain acylations on the 1-amine have shown that many are effective against both fall armyworm (*Spodoptera frugiperda* Smith) larvae and European corn borer (*Ostrinia nubilalis* Hübner) larvae, but the effects seem to differ depending on the modifications. *N*-Formylloline reduces the weight gain of fall armyworms by deterring feeding, and does not significantly affect corn borers. In contrast, NAL reduces the weight gain of corn borer larvae without changing larval feeding behavior, indicating that its effect is due to metabolic toxicity [10]. *N*-Formylloline, NAL, and NML are almost as potent as nicotine in insecticidal activity against green bugs (*Schizaphis graminum* Rondani). Furthermore, NML shows a similar regression curve to nicotine sulfate, indicating a similar pharmacological mechanism [10]. It has also been shown that lolines (mainly NFL and NAL) reduce oviposition of adult Argentine stem weevils [35]. Another study found that NFL reduces the growth, development, and survival of Argentine stem weevil larvae at high concentrations (800 and 1600 µg/g dry weight), whereas NANL causes high mortality of Argentine stem weevil larvae, but with little effect on the growth or development of the larvae [11]. These findings clearly show that the chemically diverse loline alkaloids exert complex and multimode effects on grass herbivores. Moreover, loline alkaloids also affect insect parasitoids [36], [37], thereby contributing to multitrophic endophyte effects. Thus, the diversification of loline alkaloids produced by *Epichloë* species probably reflects selection imposed by the complex and variable biotic environments of their plant hosts.

## Supporting Information

S1 Fig Chromatograms of *N*-propionylnorloline (decorticasine) and *N*-acetylloline (NAL) in loline-alkaloid-producing systems. (A) Comparison of loline alkaloids from *Adenocarpus decorticans* seed, *Epichloë uncinata* e167 in inducing culture, and meadow fescue symbiotic with e167. Only decorticasine and NAL peaks are labeled. (B) Structures of decorticasine and NAL.Chromatograms of *N*-propionylnorloline (decorticasine) and *N*-acetylloline (NAL) in loline-alkaloid-producing systems. (A) Comparison of loline alkaloids from *Adenocarpus decorticans* seed, *Epichloë uncinata* e167 in inducing culture, and meadow fescue symbiotic with e167. Only decorticasine and NAL peaks are labeled. (B) Structures of decorticasine and NAL.
(PDF)

S1 Table Oligonucleotides used in this study.
(DOCX)

S1 Protocol Construction of plasmid pKAES215.
(DOCX)
