# Supplementary material for: Enzymes from Fungal and Plant Origin Required for Chemical Diversification of Insecticidal Loline Alkaloids in Grass-Epichloë Symbiota
Source: PLoS One. 2014 Dec 22;9(12):e115590. doi: 10.1371/journal.pone.0115590 (PMC4274035; doi:10.1371/journal.pone.0115590)
Supplement: S1 Fig — Chromatograms of N-propionylnorloline (decorticasine) and N-acetylloline (NAL) in loline-alkaloid-producing systems. (A) Comparison of loline alkaloids from Adenocarpus decorticans seed, Epichloë uncinata e167 in inducing culture, and meadow fescue symbiotic with e167. Only decorticasine and NAL peaks are labeled. (B) Structures of decorticasine and NAL.Chromatograms of N-propionylnorloline (decorticasine) and N-acetylloline (NAL) in loline-alkaloid-producing systems. (A) Comparison of loline alkaloids from Adenocarpus decorticans seed, Epichloë uncinata e167 in inducing culture, and meadow fescue symbiotic with e167. Only decorticasine and NAL peaks are labeled. (B) Structures of decorticasine and NAL. (PDF) [file pone.0115590.s001.pdf]

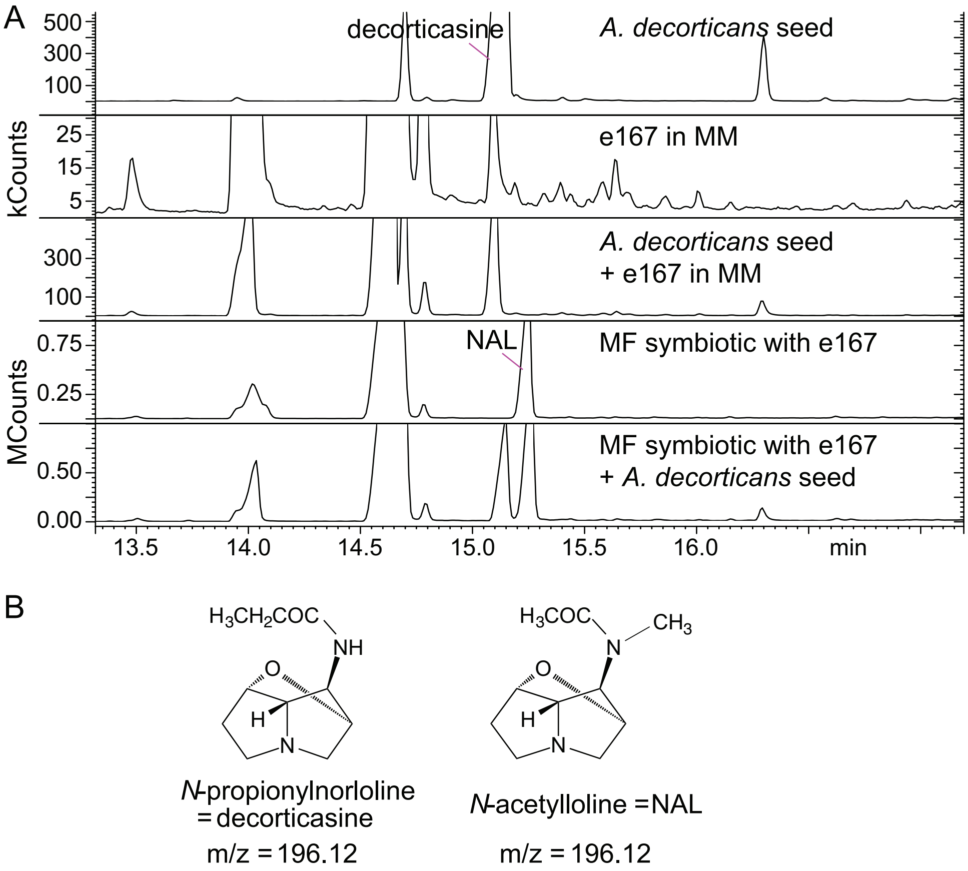

Figure S1. Chromatograms of *N*-propionylnorloline (decorticasine) and *N*-acetylloline (NAL) in loline-alkaloid-producing systems. (A) Comparison of loline alkaloids from *Adenocarpus decorticans* seed, *Epichloë uncinata* e167 in inducing culture, and meadow fescue symbiotic with e167. Only decorticasine and NAL peaks are labeled. (B) Structures of decorticasine and NAL.
