# Supplementary material for: Enzymes from Fungal and Plant Origin Required for Chemical Diversification of Insecticidal Loline Alkaloids in Grass-Epichloë Symbiota
Source: PLoS One. 2014 Dec 22;9(12):e115590. doi: 10.1371/journal.pone.0115590 (PMC4274035; doi:10.1371/journal.pone.0115590)
Supplement: S1 Table — Oligonucleotides used in this study. (DOCX) [file pone.0115590.s002.docx]

**Table S1. Oligonucleotides used in this study.**

| Primer | Sequence ^a^ |
| --- | --- |
| upFLKf | GCTCTAGATTTAAATGTTCTCCGTAAGGATAAGCATC |
| upFLKr | GCTCTAGAGTGGTGGACTGGTGAGGACT |
| lolAANMTdf | CGCCCGGGATGCACAAGGTCCAGATACATG |
| lolAANMTdr | CGCTCGAGTTATTGCTACTCTATTACCTCCT |
| aamAd1 | GCTGAGGAAGATGCGCTGAT |
| aamAup1 | CAGCGTTTAGCTGAGCACC |
| Mexpf | CGACTAGTATGACGGTGAATAGCAGCG |
| Mexpr | CTATTCCACCTTGCCCATC |
| Nexpf | CGACTAGTATGTCGAACATGAGAGCTACG |
| Nexpr | GCACGCGTCATCCAACGAGCTCCGTG |
| hphf | TCTCGTGATTCTTTCCATC |
| hphr | CGGATCGGACGATTGCGT |
| lolNMkops | ATGTAGCCCGGTGCACAGA |
| LinkerA | GATATCACGCGTCTCGAGACTAGT |
| LinkerB | ACTAGTCTCGAGACGCGTGATATC |
| MexpYNfBglII | CGAGATCTTTATGACGGTGAATAGCAGCG |
| MexpYNrBglII | CCGAGATCTCTTCCACCTTGCCCATCTTC |
| lolNampr | ACCATCTCCAAAGCGTGCAGG |

^a^ Underlined segments indicate restriction-endonuclease cleavage sites incorporated in the primers to facilitate cloning.
