# Supplementary material for: Enzymes from Fungal and Plant Origin Required for Chemical Diversification of Insecticidal Loline Alkaloids in Grass-Epichloë Symbiota
Source: PLoS One. 2014 Dec 22;9(12):e115590. doi: 10.1371/journal.pone.0115590 (PMC4274035; doi:10.1371/journal.pone.0115590)
Supplement: S1 Protocol — Construction of plasmid pKAES215. (DOCX) [file pone.0115590.s003.docx]

**Protocol S1. Construction of plasmid pKAES215.**

Plasmid pKAES215 was constructed from pKAES210, pCT74 (Lorang et al., 2001) and a polylinker. pKAES210 was constructed from pCT74 and pUC18. Plasmid pCT74 was digested with *EcoR*I and *Cla*I to produce a 1.6 kb fragment that was filled in with Klenow fragment (New England Biolabs). Plasmid pUC18 was digested with *EcoR*I and the 2.7 kb fragment was filled in with Klenow fragment. The two fragments were ligated to generate plasmid pKAES210, which has *ToxA* promoter following the *lac* promoter of pUC18. pKAES210 was then digested with *Nco*I and *Not*1. The 3.5 kb fragment was treated with Mung Bean nuclease (New England Biolabs). The polylinker was created by annealing oligonucleotides LinkerA and LinkerB together and phosphorylated by the T4 polynucleotide kinase (New England Biolabs). The polylinker was ligated with pKAES210 fragment to insert the polylinker behind the *ToxA* promoter. The resulting construct was then digested with *Sal*I and ligated to the 1.4 kb *Sal*I digested fragment of pCT74. The resulting plasmid pKAES215 contained the *ToxA* promoter followed by *Spe*I, *Xho*I, *Mlu*I and *Eco*RV restriction sites and then *nos* terminator along with *TrpC* promoter-*hph* cassette in the opposite direction of the *ToxA* promoter. Plasmid was confirmed by sequencing.

Supporting Reference:

Lorang JM, Tuori RP, Martinez JP*, et al.*, 2001. Green fluorescent protein is lighting up fungal biology. *Applied Environmental Microbiology* **67**, 1987-94.
